# Brazilian Congress of Cardiovascular Surgery

**DOI:** 10.21470/1678-9741-2017-0506

**Published:** 2017

**Authors:** Renato A. K. Kalil

**Affiliations:** 1 Instituto de Cardiologia/Fundação Universitária de Cardiologia (IC/FUC), Serviço de Cirurgia Cardiovascular, Porto Alegre, RS, Brazil.; 2 Universidade Federal de Ciências da Saúde de Porto Alegre (UFCSPA), Porto Alegre, RS, Brazil.

“As a community we should address existing gaps in outcomes with a focus on opening (rather than restricting) access to transformative technologies while lowering the barriers to educational and advanced training opportunities.

A rising tide floats all ships – but it is incumbent on us as a professional community to ensure that each of our colleagues has access to a paddle and a map. Otherwise, the resulting calamity will sink us all.”

*J. Matthew Brennan (Duke University). Ann Thorac Surg. Feb. 2016*

In the era of mass information, where is the truth?

In the era of instant information, what is a medical conference for?

These issues arise when we organize a new congress and certainly bother when the surgeon decides whether to attend an event.

In 2017, we held the 44^th^ Congress of the Sociedade Brasileira de Cirurgia Cardiovascular (SBCCV), in Rio de Janeiro, under skepticism, scarcity of resources and many doubts about the future of this activity, or even about the specialty itself.

The Congress was a success. It exceeded the expectation of the number of participants, added many new speakers and presenters of scientific subjects and impressed by the quality of the presentations of the national and international speakers. The policy adopted was to prioritize the scientific quality, to include young surgeons and to discuss the topics of the current reality and practice of the specialty. Sessions of short answers were emphasized for relevant questions, discussion of clinical trials impacting the surgical practice and presentation of original themes, both in the form of commented posters and in original oral presentations. Students and residents had their special, high-quality activities.

The 45^th^ Congress of the SBCCV will be held in Goiânia, from 19 to 21 April, 2018. The preliminary program is outlined and first line international guests are confirmed. We hope to perfect the formula, increase the focus on innovation and promote the face-to-face discussion of the topics that most concern and afflict the Brazilian cardiovascular surgeon at the moment.

On the questions put at the beginning of this editorial: where will the truth be? It can emerge from the broad, frank, qualified and face-to-face discussion, confronting data, criticism, and mixed experiences of young and experienced surgeons.

And what is a congress for? Clearly, it is the democratic opportunity to filter, from the myriad of information, what can be used and applied in surgical practice, aiming at our professional priority objective, which is the greatest benefit of the patient.

This is how SBCCV acts: understanding that one of its objectives is to promote education and access to innovation.

We look forward to your presence at our next congress.

